# Cryo-EM structure of the ATP11C Q79E mutant reveals the structural basis for altered Phospholipid recognition

**DOI:** 10.1016/j.jbc.2025.110935

**Published:** 2025-11-12

**Authors:** Yuheng Qian, Chai C. Gopalasingam, Christoph Gerle, Hideki Shigematsu, Kazuhiro Abe, Atsunori Oshima

**Affiliations:** 1 Department of Chemistry, Faculty of Science, Hokkaido University, Japan; 2 Graduate School of Pharmaceutical Sciences, Nagoya University, Nagoya, Japan; 3 RIKEN SPring-8 Center, Sayo-gun, Hyogo, Japan; 4 Japan Synchrotron Radiation Research Institute (JASRI), Sayo, Hyogo, Japan; 5 Cellular and Structural Physiology Institute (CeSPI), Nagoya University, Nagoya, Japan; 6 Institute for Glyco-core Research (iGCORE), Nagoya University, Nagoya, Aichi, Japan; 7 Center for One Medicine Innovative Translational Research (COMIT), Gifu University Institute for Advanced Study, Gifu, Japan; 8 Research Institute for Quantum and Chemical Innovation, Institutes of Innovation for Future Society, Nagoya University, Nagoya, Japan

**Keywords:** P-type ATPase, flippase, P4-ATPase, membrane protein, cryo-electron microscopy, phospholipid, transporter

## Abstract

Closely related P4-ATPases, ATP11A and ATP11C, act as major phospholipid flippases in the plasma membrane of mammalian cells, with strict substrate specificity for phosphatidylserine (PS) and phosphatidylethanolamine (PE), but not for phosphatidylcholine (PC), thereby contributing to the asymmetric distribution of PS and PE across bilayers. A previously reported disease-associated Q84E mutation in ATP11A confers the ability to flip PC, implicating the involvement of this conserved residue in substrate specificity. We performed cryo-EM analysis for the equivalent mutant Q79E of ATP11C to address the structural basis for its unusual substrate specificity. Measurement of ATPase activity revealed that the ATP11C Q79E mutant retained PS-dependent activity, whilst gaining robust PC-dependent activity, indicative of expanded substrate specificity, consistent with reported properties in ATP11A Q84E. The cryo-EM structure of ATP11C Q79E mutant in the PC-occluded E2-P_i_ state revealed a PC molecule in a reshaped binding pocket. Due to the Q79E mutation and associated conformational changes in its surrounding residues, including Ser91and Asn352, the binding pocket has additional space to accommodate the bulky choline headgroup. Our results provide structural and functional insights into how a single point mutation can alter substrate specificity in a P4-ATPase.

Biological cell membranes are composed predominantly of phospholipids. As a characteristic feature of living eukaryotic cells, these lipids are distributed asymmetrically; phosphatidylserine (PS) and phosphatidylethanolamine (PE) are confined in the cytosolic leaflet, whereas phosphatidylcholine (PC) and sphingomyelin (SM) mainly distribute in the exoplasmic leaflet (1). This asymmetry in phospholipid distribution is critical for cell integrity and physiology, as well as for regulating multiple important cellular events, including apoptosis (2, 3), which are mediated by several membrane transport proteins. One of these, the scramblases, are ATP-independent, passive membrane transporters that mediate the bidirectional, mostly nonspecific translocation of phospholipids across the lipid bilayer, along their concentration gradients. Rather than maintaining asymmetry, this activity leads to the collapse of transbilayer lipid asymmetry and is regulated by Ca^2+^ or the apoptotic protease caspase-3 (4, 5). In contrast, flippases, also known as P4-ATPases, translocate their specific substrate phospholipid from exoplasmic to the cytosolic leaflet of the bilayer coupled with ATP hydrolysis. Most mammalian P4-ATPases, except ATP9A and ATP9B (6), require an accessory subunit, CDC50A, for their proper localization and functional expression in the plasma membrane (7). The overall fold of the catalytic subunit is very similar to other P-type ATPases, composed of three cytosolic domains (N, P and A domains) involved in ATP hydrolysis. The nucleotide-binding (N) domain binds ATP. The phosphorylation (P) domain contains an invariant aspartate residue that undergoes autophosphorylation to form the phosphoenzyme intermediate (EP). The actuator (A) domain has a DGES motif that is required for the dephosphorylation of EP intermediate. The catalytic subunit also contains 10 transmembrane (TM) helices in which the phospholipid-binding site is located. The CDC50A subunit has 2 TM helices at the N and C termini and a large exoplasmic domain.

Among P4-ATPases, the closely related ATP11A and ATP11C are particularly important because of their involvement in apoptosis (3). In living cells, PS is largely maintained on the cytosolic leaflet of the membrane. Cells that lack ATP11A, ATP11C, or the accessory subunit CDC50A exhibit a significant reduction in flippase activity for PS and PE at the plasma membrane, indicating that ATP11A and ATP11C are major PS-specific flippases in the plasma membrane (8). When a cell undergoes apoptosis, an activated caspase cleaves ATP11A and ATP11C, causing inactivation of their ability to translocate PS. Simultaneously, caspase-dependent scramblases such as Xkr8 (9) or Xkr4 (5) are activated, which expose PS on the exoplasmic leaflet of the plasma membrane. This exposure of PS acts as an “Eat-me” signal, marking the dying cell for engulfment by macrophages. Mice deficient in ATP11A do not survive embryonic development (10), and mice lacking ATP11C display a variety of complex phenotypes, including B-cell lymphopenia, cholestasis, mild anemia, and dystonia (11). Therefore, understanding the mechanism of ATP11A and ATP11C is crucial in comprehending the process of cell death at a molecular level.

In cation-transporting P2-type ATPases, the transported cations interact with residues located in TM4, TM5, TM6, and TM8 (12, 13, 14). These residues are situated in the central region of the transmembrane domain and are shielded from the lipid phase by surrounding helices. During the transport cycle, cations are occluded between these central helices (15, 16). However, in the case of flippases, due to the larger size of lipid substrates and the need for reorientation during translocation, a distinct transport mechanism has been proposed—the so-called "credit-card model" (17). In this model, phospholipid translocation is thought to occur at the protein–lipid interface near the periphery of the transmembrane domain, where only the hydrophilic lipid headgroup interacts with the protein, while the acyl chains are dragged through the membrane bilayer (18). To explain this process in more detail, three models have been proposed based on studies of different P4-ATPases, including the two-gate model (19), the hydrophobic gate model (20), and the central cavity model (21), together which suggests that phospholipid flipping is achieved through sequential conformational changes in the transmembrane helices, which alternately open and close toward the outer and inner leaflets to control substrate access and release.

Nevertheless, as a member of the P-type ATPase family, the transport cycle of P4-ATPase generally resembles that of other P2-type cation pumps (Fig. 1*A*), often referred to as the E1/E2 model, or, Post-Albers type reaction scheme (22, 23, 24), proceeding through reaction intermediates including E1, E2, and their autophosphorylated forms E1P and E2P. Unlike P2-ATPases, P4-ATPases have evolved distinct structural mechanisms to selectively translocate phospholipids across the membrane, which has been extensively studied through structural analysis, including that of the yeast flippase Drs2p (25) and human flippase ATP8A1 (26). As for human ATP11C, multiple cryo-EM structures capturing different intermediate states have delineated a near complete flipping cycle, from the E1-ATP to the E2-P_i_ state, explaining molecular mechanisms of how PS occlusion induces E2P dephosphorylation (27). According to the transport mechanism of P4-ATPase (Fig. 1*A*), PS is incorporated from the exoplasmic leaflet of the bilayer to the binding site in the outward-open E2P state. The terminal carboxyl group of bound PS subsequently induces outer gate closure by forming hydrogen bonds with several amino acids in exoplasmic parts of TM1 and TM2 that constitute the outer gate. The outer gate closure is transmitted to the A domain and changes the relative orientation between A and P domains, leading to a transition state of E2P dephosphorylation, E2-P_i_ state, in which PS is occluded in the TM binding site. Consequently, these two E2P-related intermediates are key to elucidating the mechanism of phospholipid recognition.

**Figure 1:**
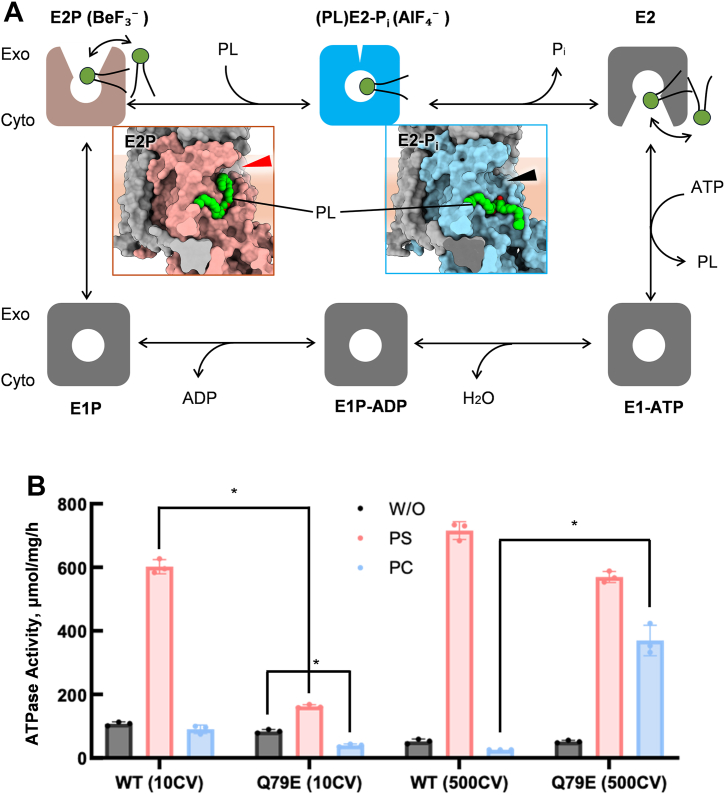
**Reaction scheme and phospholipid (PL)-dependent ATPase activity of ATP11C.***A*, the cycle consists of four principal conformational states: E1 (not show in the figure), E1P, E2P, and E2, where “P” denotes a covalently bound phosphate group. E2P reflects the phosphorylated state prior to substrate binding, while E2-P_i_ captures the enzyme conformation after substrate binding but just before phosphate release. The models in surface representation show the outward-open E2P state (*brown box*, *left*) and PL-occluded (PL)E2-P_i_ state (*blue box*, *right*). Phospholipids are shown as *green spheres*. The *red arrowhead* indicates the outward gate exposed to the exoplasmic leaflet in E2P state, while it is closed in the PL-occluded E2-P_i_ state (*black arrowhead*). *B*, sSpecific ATPase activity of ATP11C WT and Q79E mutant measured in the absence of phospholipid (W/O, *grey*) and presence of 100 μM DOPS (PS, red) or DOPC (PC, *blue*) added to the sample with 10 or 500 column volume (CV) wash. The value obtained from the sample with 1 mM BeF_3_^-^ serves as a blank. Data plotted are mean ± S.D. from three independent experiments. *Asterisks* indicate the *p* values between the indicated data sets are ≦ 0.05.

Recently, the Gln84Glu (Q84E) mutation in ATP11A was identified in a patient with developmental delay and neurodegeneration. Surprisingly, this mutant confers the ability to translocate PC in addition to the canonical substrate PS. This aberrant substrate specificity leads to an accumulation of sphingomyelin (SM) on exoplasmic leaflet of the plasma membrane, thereby altering various cellular properties, including cell growth, cholesterol homeostasis, and sensitivity to sphingomyelinase (28). It was also reported that its equivalent mutation Gln79Glu (Q79E) in ATP11C shows PC translocation in living cells (28), suggesting the mechanisms for substrate recognition are preserved in these two closely related flippases (sequence identity of 64%, Fig. S1). However, the mechanism by which aberrant substrate recognition occurs in this disease mutant remains unclear. To reveal the structural basis underlying this abnormal substrate recognition, we determined the cryo-EM structure of ATP11C Q79E mutant in the PC-occluded state. The results suggest that unusual PC recognition in the Q79E mutant is, counterintuitively, not due to enhanced electrostatic interactions, but rather an indirect change in the shape of the binding pocket resulting from the mutation. This finding deepens our understanding of the substrate recognition mechanism by P4-ATPases, providing further insight into P-ATPase-related diseases and potential therapeutic strategies.

## Results and discussion

### Functional characterization of the ATP11C Q79E mutant

A previous report showed that ATP11C Q79E, a mutation equivalent to a disease-causing variant of ATP11A Q84E, transports PC in addition to its canonical substrate, PS (28). To confirm this altered substrate specificity, we expressed and purified both wild-type (WT) and Q79E ATP11C and compared their phospholipid-dependent ATPase activity (Fig. 1*B*). The WT enzyme showed high ATPase activity in the presence of PS but only background levels (no added lipid) in the presence of PC, consistent with its PS-specific ATPase activity (27). Unexpectedly, however, the ATP11C Q79E mutant showed significantly lower activation by PS compared to the WT, while the addition of PC resulted in the activity being even lower than the background level. This observation prompted us to hypothesize that the excess amount of PC or other phospholipids showing an inhibitory effect may be left over in the sample. We speculated that this inhibition is related to the reverse reaction occurred in the cytoplasmic facing state. According to the Post-Albers type scheme (Fig. 1), substrate phospholipid is released to the cytoplasmic leaflet during E2→E1 transition, excess amount of substrate may accelerate E1→E2 reverse reaction that apparently reduces ATPase activity. Since the samples used for the above measurements were purified with anti-GFP nanobody affinity resin, which included a washing step with washing buffer of only 10 times the column bed volume (10CV), we therefore attempted to remove the remaining phospholipids by performing a more rigorous washing (500CV). The ensuing samples showed that both WT and Q79E mutant were activated by PS, and only the Q79E mutant shows significant activation upon PC addition (Fig. 1*B*). Given that PC transport by ATP11C Q79E has been experimentally demonstrated in a previous report (28), the PC-dependent activation is unlikely to result merely from nonspecific stabilization by annular lipids. This PC-dependent activation suggests that exogenously added PC interacts with the binding site of the Q79E mutant sample purified with extensive (500CV) washing.

### Cryo-EM structure of PC-bound E2P state

**Figure 2:**
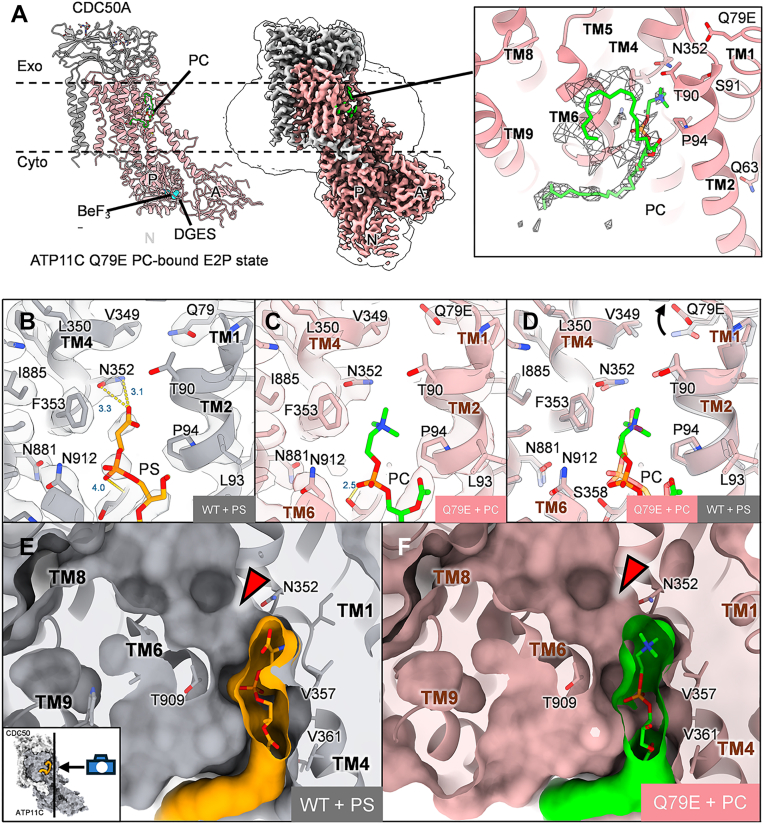
**Cryo-EM structure of ATP11C Q79E in E2P state.***A*, the atomic model (*left*) and cryo-EM map (*middle*) of ATP11C Q79E E2P PC-bound state. Bound PC in the TM region is shown as *green sticks*, and its close-up view with a density map (only showing within 4 Å from the PC model in mesh representation) is shown on the *right*. A-, P-, N-domains and DGES motif are indicated in the figure. BeF_3_^-^ are shown as cyan spheres. *B–D*, close-up view of phospholipid binding site of E2P state for WT (*B*) and Q79E mutant (*C*) and their comparison (*D*), viewed parallel to the membrane plane, from above the exoplasmic side. Cryo-EM density maps (transparent surface) and fitted atomic models of PS-bound ATP11C WT (*B*, PDB: 7BSU) and PC-bound ATP11C Q79E (*C*) are shown. *Yellow dotted lines* in *B* and *C* indicate probable hydrogen bond or salt bridge (with distance less than 4 Å) involved in phospholipid coordination. The arrow in *D* indicates the different rotamer conformation between WT and Q79E. *E* and *F*, cross-sectional surface representations of the lipid-binding pocket for WT (*E*) and Q79E (*F*). Phospholipids (*sticks*) are shown with their van der Waals surfaces displayed, for PS (*orange*) and PC (*green*), from the viewpoint where TM2 is located. *Red arrowheads* in both *E* and *F* show the unoccupied part of the binding pocket, where the pocket doesn’t tightly constrain the phospholipid headgroup. A small inset in *E* shows the viewing angle used for the main snapshot, as context for the orientation of the binding pocket shown in panels *E* and *F*.

Using the above-described samples purified with extensive washing, we performed cryo-EM structural analysis (Figs.2, S2, S3, and Table S1). We first determined the structure of the outward-open E2P state, at 2.88 Å resolution, stabilized by the phosphate analog BeF_3_^-^ and exogenously added DOPC, to confirm whether the mutation itself perturbs the conformation or binding site structure. A structural comparison between WT (PS-bound E2P state, 7BSU) and Q79E reveals that these structures are superimposable (RMSD = 0.56 Å) (Fig. S4), confirming that the mutation does not significantly affect the overall molecular conformation. A density corresponding to a bound phospholipid, most likely PC, was observed in the same position as in the WT; deep within a longitudinal cleft that extends from the exoplasmic leaflet to the canonical phospholipid binding site where TM4 is unwound (Figs. 1*A* and 2*A*). This density resolved the acyl chains but was more diffuse for the hydrophilic head group, suggesting that the phospholipid is not tightly bound (Fig. 2, *A* and *C*). In the E2P state of the WT, PS forms only a few hydrogen bonds with the binding site, including an interaction between Asn352 in the unwound region of TM4 and the terminal carboxyl group of PS (Fig. 2*B*). This indicates that the WT binding pocket in the E2P state loosely accommodates the PS head group, and this is also the case for the bound PC in the Q79E structure (Fig. 2*C*). In addition, since the terminal methyl group of PC’s quaternary amine cannot form a hydrogen bond with Asn352 of Q79E mutant, PC binding is likely much weaker than PS in the WT, as seen by its weak density (Fig. 2*C*). Although the side chain of Q79E was slightly offset relative to that of Q79 in WT (Fig. 2*D*), as the Q79E residue is located quite distant (10.3 Å) from the PC head group, making it unlikely to be directly involved in substrate recognition in the E2P state. Because the observed structure is in the outward-open state, the binding pocket is not constricted enough to restrict PC’s binding pose, possibly explaining the reason for the weak EM density for bound PC in the Q79E mutant (Fig. 2, *E* and *F*).

### Local remodeling of the substrate-binding pocket by Q79E facilitates phosphatidylcholine recognition

**Figure 3:**
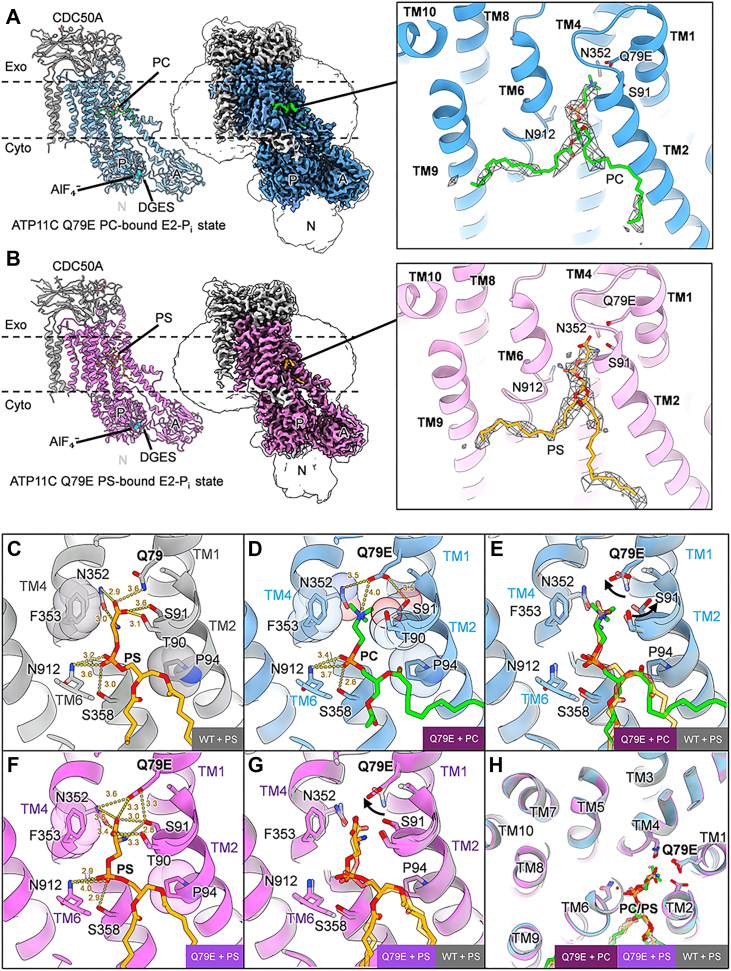
**Cryo-EM structure of ATP11C Q79E in E2-P_i_ state.***A* and *B*, the atomic model (*left*) and cryo-EM map (*middle*) of ATP11C Q79E E2-P_i_ state with occluded PC (*A*) or PS (*B*). PC and PS in the TM region are shown as *green* and *orange sticks*, respectively, and their close-up views are as shown in Figure 2*A*. AlF_4_^-^ is shown as *cyan spheres*. *C–H*, c-up view of phospholipid binding site of E2-P_i_ state for WT (*C*, PDB: 7BSV), Q79E mutant with PC (*D*) and Q79E with PS (*F*) and their comparison (*E*, *G*, and *H*) as shown in Figure 2. Residues in transparent sphere representations are located within 3.5 Å from the occluded phospholipids, likely involved in van der Waals contact. Comparison of TM helices between ATP11C WT with PS (*gray*), ATP11C Q79E with PC (*blue*) and PS (*pink*), is viewed from the exoplasmic side for (*H*), while close-up views from parallel to the membrane plane are shown for PS-WT and PC-Q79E (*E*), and PS-WT and PS-Q79E (*G*). *Arrows* indicate different rotamer conformation between WT and Q79E.

After the phospholipid binds to the E2P state, the hydrophilic head group of the substrate phospholipid is tightly occluded, accompanied by the closure of the outer gate composed of the extracellular portion of TM1 and TM2. This conformational change triggers the rotation of the A domain *via* TM1 and TM2, which in turn causes dephosphorylation of E2P (Fig. 1*A*). In ATP11C WT, the head group of PS forms hydrogen bonds with hydrophilic amino acids in TM1 and TM2 that include Gln79, Thr90 and Ser91 in the PS-occluded E2-P_i_ state (PDB ID: 7BSV), which is proposed to be a driving force for the gate closure and accompanying E2P dephosphorylation (27). Accordingly, PC-dependent ATPase activity observed in the Q79E mutant (Fig. 1*B*) requires outer gate closure driven by PC binding. We next determined the structures of PC- and PS-occluded E2-P_i_ state, analyzed at 2.40 Å and 2.51 Å, respectively, to address how PC is recognized in the occluded binding pocket (Figs. 3, S2, S3, and Table S1).

Cryo-EM density maps showed well-resolved amino acid side chains surrounding the phospholipid binding site (Fig. S5). Although the density of the bound phospholipid itself was not of sufficient quality to identify it as either PC (Figs. 3*A* and S5*B*) or PS (Figs. 3*B* and S5*C*), since the ATPase activity of the purified mutant sample is largely stimulated by PC addition (Fig. 1*B*), it is likely that an exogenously added phospholipid is bound to the site. Arrangement of TM helices, especially TM1, TM2, TM4 and TM6 that form the phospholipid-binding pocket, is unchanged between PS-occluded WT and PC-occluded Q79E mutant (Fig. 3*H*), suggesting a preserved gating mechanism between them. The PS head group is coordinated by several key hydrogen bonds in the PS-occluded form of Q79E mutant (Fig. 3*F*), which is very similar to WT (Fig. 3*C*), consistent with its PS-dependent ATPase activity. In contrast, PC, with three methyl groups at its terminus, cannot form hydrogen bonds with surrounding hydrophilic side chains. The nitrogen of the quaternary amine in PC is located more than 4 Å from the carboxyl group of Q79E (Fig. 3*D*), suggesting that it does not engage in strong electrostatic interactions. This observation refutes an intuitive hypothesis that the Gln to Glu mutation enhances PC binding due to increased electrostatic interaction, as the nitrogen on the PC quaternary amine is presumed to be positively charged at a neutral pH.

**Figure 4:**
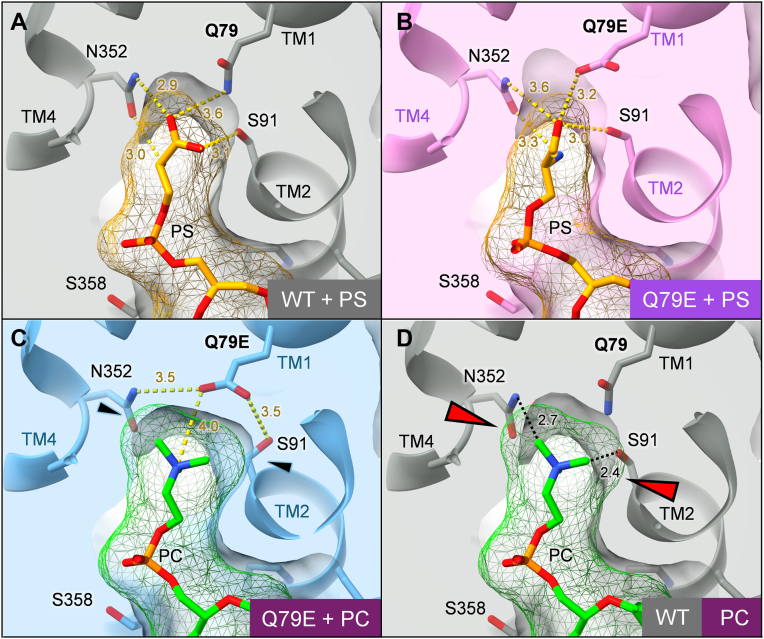
**Phospholipid-occlusion in the binding pocket of ATP11C.** Cross-sectional surface representations of the lipid-binding pocket in ATP11C WT with PS (*A*) and Q79E with PS (*B*) or PC (*C*) in E2-P_i_ state. For comparison, only the PC molecule in Q79E mutant is superimposed on the WT structure in D. Phospholipids are shown as sticks with their van der Waals radii in mesh representation. *Black arrowheads* in *C* indicate residues with altered rotamer conformation due to the Q79E mutation. *Red arrowheads* indicate expected steric clash between PC and the WT binding pocket.

A detailed comparison of the WT and Q79E mutant structures revealed that the respective side chain rotamer conformations (*i.e.*, Q79 in WT and E79 in Q79E mutant) are different (Fig. 3, *E* and *G*), each rotating approximately 90° relative to one another. The corresponding density map at this position well supports the rotamer conformation of these residues (Fig. S5). We found that this observed difference likely affects the surrounding amino acids in the binding site. In the WT structure, the hydroxyl group of the Ser91 residue is fixed by an interaction with the Gln79 side chain, forming a hydrogen bond, and thus Ser91 side chain is pointing toward the carboxyl group of PS (Fig. 3*C*). In contrast, the Ser91 side chain in the Q79E mutant faces the opposite direction, and it appears to stabilize its conformation by forming a hydrogen bond with the carboxyl of Q79E (Fig. 3*E*, see arrow for the displacement of side chains). Consequently, C_β_ carbon of Ser91 is located 3.4 Å from the terminal methyl group of PC. Due to the rotation of Q79E relative to Gln79 in WT, Asn352 makes a hydrogen bond with Q79E, thus the side chain conformation of Asn352 also slightly changes compared to that in WT. Accumulation of these subtle changes in the rotamer conformation results in an altered shape of the binding site in Q79E (Fig. 4). While the WT binding pocket and PS-occluded structure of the Q79E mutant are ideally shaped to fit the PS head group (Fig. 4, *A* and *B*), the PC-occluded structure of the Q79E mutant shows a slightly widened pocket, particularly due to the changes in Ser91 and Asn352 side chain conformations that directly contact PC head group (Fig. 4*C*). We found that this widening is crucial for accommodating the PC head group. When superimposing the PC molecule in Q79E structure against the WT structure, the terminal methyl groups of PC would sterically clash against Asn352 and Ser91(Fig. 4*D*). These observations lead us to conclude that the ability of the Q79E mutant to bind PC is a result of subtle, indirect conformational changes in the surrounding amino acids, which cause local reshaping of the binding site to optimally accommodate PC. van der Waals and entropy-driven hydrophobic interactions act as the primary driving force for PC to fit into this newly shaped pocket, triggering the closure of the exoplasmic gate, which leads to PC-dependent dephosphorylation, as discussed later.

### Structural and functional characterization of the Q79A mutant

**Figure 5:**
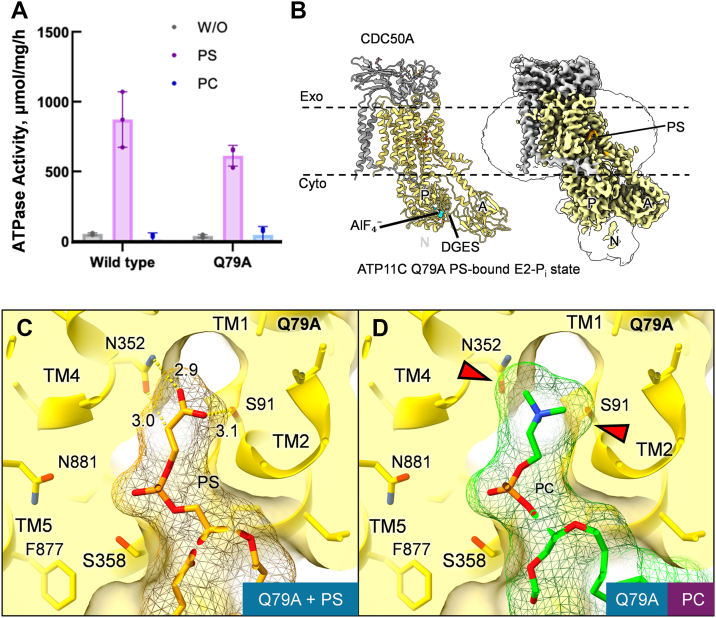
**Cryo-EM structure of ATP11C Q79A in E2-P_i_ state.***A*, specific ATPase activity of ATP11C Q79A mutant sample purified with 500 CV as in Figure 1*B*. Data plotted are mean ± S.D. from three independent experiments. *B*, the atomic model (*left*) and cryo-EM map (*right*) of ATP11C Q79A PS-occluded E2-P_i_ state as in Figure 2. *C*, cross-sectional surface representations of the lipid-binding pocket in ATP11C Q79A as in Figure 4. *D*, the PC molecule observed in the structure of Q79E in PC-occluded E2-P_i_ state was superimposed onto the Q79A lipid-binding pocket as in Figure 4*C*.

If the size of the binding pocket is a key determinant in PC binding, the mutation of Gln79 to a smaller residue, like alanine, would allow PC binding. At least for ATP11A, this is not the case, as its Gln84Ala mutant does not exhibit PC translocation activity (28). To investigate this point further, we evaluated the Gln79Ala (Q79A) mutant of ATP11C. Consistent with the equivalent mutant of ATP11A, ATP11C Q79A retained PS-dependent ATPase activity, but not for PC (Fig. 5*A*), indicating that PC cannot be occluded in the binding pocket of this mutant.

To help reveal the molecular framework for this, we solved the cryo-EM structure of ATP11C Q79A mutant at 3.44 Å resolution (Fig. 5*B*, see also Figs. S2, S3 and Table S1). Due to the smaller side chain of Q79A, the binding pocket becomes larger in the direction toward TM1 (Fig. 5*C*). However, because the Ser91 hydroxyl group remains facing toward the phospholipid binding site, similar to that in WT structure, the shape of the pocket is not widened (*i.e.*, unchanged) towards TM2 or TM4. When we superimposed the occluded PC in Q79E mutant against the Q79A structure, the binding pocket in Q79A mutant is not wide enough to accommodate PC, due to unaltered rotamer conformation of Ser91 compared to that of WT (Fig. 5*D*). These observations suggest that the hydroxyl group of Ser91 is inherently oriented toward the phospholipid binding site, and an additional hydrogen bond contribution from Q79E side chain may stabilize it in the opposite direction (Fig. 3*D*), which is a key requisite for the widened binding pocket found in the Q79E mutant (Fig. 4*C*).

### Structural comparison with ATP8B1

ATP8B1 was initially identified as the gene responsible for progressive familial intrahepatic cholestasis type 1 (PFIC1). It is proven that ATP8B1 has a broad lipid specificity, including PC, PS and phosphatidylinositol (PI) (29). Sequence alignment of ATP8B1 to ATP11C shows a high degree of conservation at the lipid-binding cavity (Fig. S1). To explore the common features of such flippases with broader specificity and to test whether our proposed mechanism of phospholipid recognition can be applicable to other flippases, we compared the structures of ATP8B1 and ATP11C.

**Figure 6:**
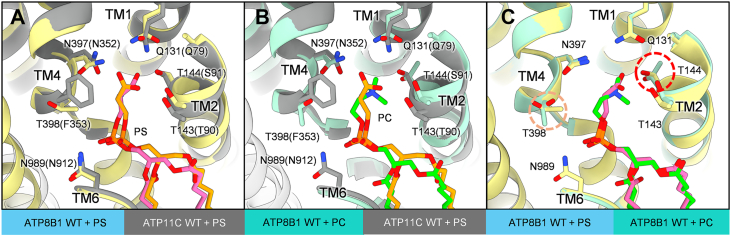
**Structural comparison with PL-occluded ATP8B1.***A–C*, comparison of the phospholipid binding models in PS-occluded ATP8B1(8OXA, *yellow*), PC-occluded ATP8B1 (8OXB, *salmon*) and PS-occluded ATP11C WT (7BSV, *gray*) as indicated in figures. Only amino acids close to the phospholipid binding site are shown as sticks. Amino acids and their numbering are indicated according to ATP8B1, and their corresponding residues in ATP11C WT are displayed in parentheses. PS from ATP8B1 is colored *pink*, while PS from ATP11C WT is colored *orange*. *Dashed circles* in C indicate residues with different rotamer conformations between PS- and PC-occluded forms of ATP8B1.

In the phospholipid-occluded E2-P_i_ state (Fig. 6*A*), ATP8B1 and ATP11C WT share similar spatial organization of the transmembrane helices (TM1, TM2, TM4 and TM6) that form the binding pocket. Accordingly, the structure of the side chains around the binding pocket likely determines which phospholipid can bind. Due to its broad ligand specificity, both PS- and PC-occluded structures are available for ATP8B1 (PDB IDs 8OXA and 8OXB for PS- and PC-occluded form, respectively). Intriguingly, in ATP8B1 WT, the Thr144 side chain (which corresponds to Ser91 in ATP11C) adopts a different rotamer conformation when comparing the PC-occluded state to the PS-occluded state (Fig. 6). This behavior of Thr144 in ATP8B1 is well correlated with what was seen in our PS-occluded ATP11C WT (PS, Fig. 3*C*) and its Q79E mutant with PC- (Fig. 3*D*) or PS-occluded (Fig. 3*F*); the hydroxyl group of Thr144 is pointing toward the carboxyl group of PS (Fig. 6*A*), while it is facing toward the other side in the PC-occluded form (Fig. 6*B*). Although ATP8B1 has a glutamine residue in TM1 which corresponds to Gln79 in ATP11C, its rotamer conformation is similar to that seen in the Q79E mutant of ATP11C (Fig. 3*E*). These similarities between two flippases suggest a common mechanism for PC accommodation within the binding pocket.

### Driving force for the exoplasmic gate closure

Based on previous structural analysis of the WT ATP11C transport cycle, it is thought that the interaction between the hydrophilic PS head group and several amino acids that consist of the outer gate serves as a driving force for gate closure. This process acts as a checkpoint: phospholipids that are too large to be occluded, or those that cannot sufficiently stabilize the occluded state, cannot advance the transport cycle to the next step, thus preventing either dephosphorylation or subsequent lipid translocation. In contrast to PS, which has terminal carboxyl and amino groups, the choline head group of PC, with its positively charged quaternary amine and three methyl groups, is less capable of forming strong hydrophilic interactions. Consequently, the driving force for gate closure induced by PC binding would be predominantly *via* hydrophobic interactions. The general physicochemical principle of entropy-driven or hydrophobic ligand-binding is that the close packing of a ligand within its binding pocket displaces hydrated water molecules from the vicinity, leading to a favorable increase in entropy of the overall system. In other words, a tight-binding pocket shows stronger binding energy. Therefore, if PC cannot utilize hydrogen bonds to drive gate closure, it must acquire sufficiently strong alternative hydrophobic interactions to compensate. Our observed reshaping of the binding pocket in the Q79E mutant, thus, likely creates a suitable environment to drive the hydrophobic interaction (Figs. 3, and 4*C*). If size matters, phospholipids with smaller head groups should theoretically be able to be translocated. We therefore conclude that the subtle, indirect conformational change initiated by the Q79E mutation creates a binding pocket that precisely fits the shape of the PC head group (Fig. 4*C*), which provides sufficient binding energy upon PC occlusion, thus facilitating the forwarding of the transport cycle.

## Conclusion

**Figure 7:**
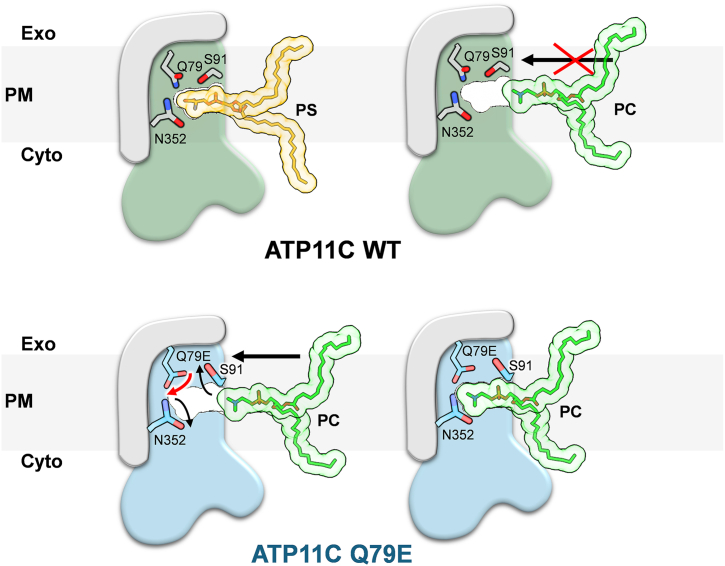
**A model of PC recognition in ATP11C Q79E.** In the ATP11C WT (*top*), canonical substrate PS is recognized by hydrogen bonds in its occluded form, and the binding pocket fits well to the head group of PS, which is too narrow to accommodate PC. In contrast, given the different rotamer conformation of Q79E (*red arrow*), the binding pocket in Q79E (*bottom*) is widened due to subsequently altered rotamer conformations of Ser91 and Asn352 (*black arrows*), thus allowing PC occlusion.

In this study, we revealed how a single point mutation of Q79E in ATP11C alters its substrate specificity by reshaping the local architecture of the phospholipid-binding pocket. Our analysis for PC-dependent ATPase activity, together with a previous phospholipid transport assay (28) demonstrated that the Q79E mutant gains the ability to recognize and flip PC, a lipid not utilized by the wild-type enzyme. Structural analyses by cryo-EM uncovered that this gain of function arises from subtle but critical rearrangements of side chains—particularly Glu79 and Ser91—that expand the binding cavity and change the interaction network, suggesting that such physicochemical compatibility within the lipid-binding pocket acts as a predeterminant for substrate selection (Fig. 7).

Furthermore, recent studies have shown that two *de novo* ATP11A mutations near the putative phospholipid exit site (E114G and S399L) also enable flipping of PC (30). These mutations—along with the Q79E mutation in our study, which is located near the substrate entry site—collectively demonstrate that structural alterations at both ends of the translocation pathway can modulate lipid specificity. Similar mutations with altered substrate specificity have been reported in ATP8A2 and Dnf2p (31, 32, 33). The cryo-EM analysis of these mutants could also help shed light on how the exit site mutations affect the overall architecture of the binding pocket.

## Experimental procedures

### Protein expression and purification

Protein expression procedures were performed as previously described (34) (Nakanishi *et al.*, 2020). In brief, human ATP11C (NCBI: XM_005262405.1) was subcloned into a custom-made expression vector. To facilitate structural studies, the N-terminal 7 amino acids (ΔN7) and the C-terminal 38 amino acids (ΔC38) of ATP11C were truncated. An N-terminal fusion tag containing a FLAG epitope (DYKDDDDK), a hexahistidine tag (His_6_), enhanced green fluorescent protein (EGFP), and a tobacco etch virus (TEV) protease recognition sequence was inserted upstream of the truncated ATP11C (referred to as ATP11C_cryst). Mutants Q79E and Q79A were generated by site-directed mutagenesis using the ATP11C_cryst construct as a template.

Human CDC50A cDNA (NCBI: NM_018247.3) was subcloned independently into the same vector system. To regulate its glycosylation state, a double mutation (Asn190Gln and Ser292Trp) was introduced into CDC50A, yielding the CDC50A_QW variant. The N190Q mutation prevents N-linked glycosylation, while the S292W mutation eliminates O-linked glycosylation at Ser292 and enhances N-linked glycosylation at Asn294, as these sites are known to be alternatively glycosylated.

Recombinant baculoviruses were generated using Bac-to-bac system and amplified in Sf9 suspension culture maintained in SF900II medium (Thermo) supplemented with 0.5% Penicillin-Streptomycin-Amphotericin B (Wako). Expi293F cells (Thermo) were maintained in Expi293 medium (Thermo) supplemented with 0.5% Penicillin-Streptomycin-Amphotericin B (Wako). Cells used for protein expression were cultured in FreeStyle293 medium (Thermo) supplemented with 0.5% FBS, 0.5% Penicillin-Streptomycin-Amphotericin B (Wako), 4 mM L-alanyl-L-glutamine (Wako).

The heterodimer composed of ATP11C_cryst and CDC50A_QW (here referred as wild-type) was successfully expressed in the plasma membrane using baculovirus-mediated transduction of mammalian Expi293 cells (Thermo) for 24 h at 37 °C in the presence of 10 mM sodium butyrate as described previously (35, 36). The harvested cells were directly solubilized with 1.5% (w/v) n-decyl β-D-maltoside in a lysis buffer containing 40 mM MES/Tris (pH 6.5), 200 mM NaCl, 2 mM Mg(CH3COO)_2_, 1 mM ATP, 1 mM dithiothreitol, 0.1 mM ethylene glycol-bis(2-aminoethylether)-N,N,N′,N′-tetraacetic acid (EGTA) and protease inhibitor cocktail (Roche) on ice for 20 min. After removing the insoluble material by ultracentrifugation (200,000×*g* for 1 h), the supernatant was mixed with anti-GFP nanobody resin (37) at 4 °C for 2 h, which was followed by washing with 500 column volumes (28) buffer consisting of 20 mM MES/Tris (pH 6.5), 200 mM NaCl, 5% (v/v) glycerol, 1 mM MgCl_2_, 0.1 mM ATP, 0.1 mM EGTA and 0.03% octaethylene glycol monododecyl ether (C_12_E_8_, Nikko Chemical), in the presence of 1 mM BeSO_4_ and 3 mM NaF (E2P state) or 1 mM AlCl_3_ and 5 mM NaF (E2-P_i_ state), and 10 column volumes washing with buffer consisting of 20 mM MES/Tris (pH 6.5), 200 mM NaCl, 5% (v/v) glycerol, 1 mM MgCl2, 0.1 mM ATP, 0.1 mM EGTA, 30 μM phospholipid (DOPS or DOPC), 2 mM BeSO_4_ and 6 mM NaF (E2P state) or 2 mM AlCl_3_ and 10 mM NaF (E2-P_i_ state), and 0.06% glyco-diosgenin (GDN) (20). After addition of TEV protease and endoglycosidase, anti-GFP nanobody was incubated at 4 °C overnight. Digested peptide fragments containing EGFP and endoglycosidase were removed by passing the fractions through Ni-NTA resin (Qiagen). Flow-through fractions were concentrated and subjected to size-exclusion column chromatography using a Superose 6 Increase column (Cytiva) equilibrated in a buffer comprising 20 mM MES/Tris (pH 6.5), 1% (v/v) glycerol, 50 mM NaCl, 5 mM MgCl_2_, 30 μM phospholipid (DOPS or DOPC), 2 mM BeSO_4_ and 6 mM NaF (E2P state) or 2 mM AlCl_3_ and 10 mM NaF (E2-P_i_ state), and 0.06% GDN. Peak fractions were collected and concentrated to 8 mg/ml. For the ATPase measurement, anti-GFP nanobody resin was washed with a buffer containing 0.03% C_12_E_8_ and 0.1 mM ATP in the absence of phospholipid and BeF or AlF. After overnight treatment with TEV protease and endoglycosidase, following Ni-NTA, the sample was concentrated to 1 mg/ml and subjected to the ATPase measurement as described later. Note that the effect of truncation of both terminal amino acids of ATP11C, the double mutation introduced to CDC50A and deglycosylation of CDC50A on the PS and PE dependent ATPase activity was negligible compared with the unmodified enzyme (34).

### ATPase activity assay

ATPase activity assay was performed as described previously (27) with modifications. Partially purified ATP11C (WT or mutants), which was enriched by affinity purification without further SEC fractionation, was used for activity measurements. Briefly, after solubilization of cell membranes and mixing with anti-GFP nanobody resin, the sample was washed with 500 column volumes of buffer containing 0.03% C_12_E_8_ and 0.1 mM ATP in the absence of phospholipid and BeF or AlF. After overnight treatment with TEV protease and endoglycosidase, followed by Ni-NTA, the sample was concentrated to 1 mg/ml and subjected to the ATPase measurement. For the measurement, the sample was diluted to 2 μg/ml and suspended in buffer comprising 40 mM MES/Tris (pH 6.5), 2 mM MgCl_2_, 2 mM ATP, 2% glycerol, 100 mM NaCl, 0.03 mg/ml of C_12_E_8_ in the absence and presence of 100 μM phospholipids (DOPS or DOPC, dissolved as 10 mg/ml of stock in 2% C_12_E_8_), in 96-well plates. We added 1 mM BeSO_4_ and 3 mM NaF (BeF_3_^−^) to every sample as a blank. Reactions were initiated by incubating the samples at 37 °C using a thermal cycler, and maintained for 1 h. It was confirmed that in a 1-h incubation with 2 mM ATP, approximately 15% of the total ATP was hydrolyzed, indicating that ATP hydrolysis is carried out within the linear range during the reaction. Reactions were terminated by adding 1% SDS and 2 N HCl, and the amount of inorganic phosphate released was determined colorimetrically using a microplate reader (Thermo).

### Cryo-EM grid preparation

Cryo-EM grids were prepared as described (26) with modifications. The purified protein sample (final protein concentration of 8∼10 mg/ml) was mixed with 12 μM DOPS or 12 μM DOPC and applied to freshly glow-discharged (double side treatment) Quantifoil holey carbon grids (R1.2/1.3, Cu/Rh, 200 mesh), using a Vitrobot Mark IV (Thermo Fisher) at 4 °C with a blot force of 10 and blot time of 4 s under 100% humidity, and then plunge-frozen in liquid ethane.

### Cryo-EM data Collection and image Processing

The prepared grids were transferred to a CRYO ARM 300 microscope (JEOL), operated at 300 kV, with a cold-field emission gun as the electron source, an in-column Omega filter and equipped with a Gatan K3 direct electron detector in the electron counting mode. Imaging was performed at a nominal magnification of 600,00 ×, corresponding to a calibrated pixel size of 0.752 Å/pix (EM01CT at SPring-8). Each movie was recorded in correlated-double sampling (CDS) electron counting mode for 2.6 s and subdivided into 60 frames. The electron flux was set to 8.46 e^−^/pix/s at the detector, resulting in an accumulated exposure of 60 e^−^/Å^2^ at the specimen. The data were automatically acquired by the beam-image shift method using SerialEM software, version 7.8 (38), with a defocus range of −1.4 to −1.6 μm. The dose-fractionated movies were subjected to beam-induced motion correction, using RELION 3.1 (39), and the contrast transfer function (CTF) parameters were estimated using patch CTF estimation in cryoSPARC (40).

For each dataset, particles were initially picked by blob picker using cryoSPARC (v4) and extracted with down-sampling to a pixel size of 3.008 Å/pix. These particles were subjected to several rounds of 2D classifications. Selected classes were then subjected to *ab initio* reconstruction in three models and refined by non-uniform refinement. The particles from the best class were then re-extracted to the full pixel size and subjected to non-uniform refinement with per-particle defocus refinement, beam-tilt refinement in cryoSPARC (40). The resolution of the analyzed map was defined according to the FSC = 0.143 criterion (41). The local resolution and angular distributions for each structure were estimated by cryoSPARC (v4). All the models were manually built in Coot using PS-bound ATP11C-CDC50A WT E2P state (7BSU) or E2-Pi state (7BSV) as a template (27). Phenix (version 20) (42) was used for refinement.

## Data availability

The following atomic models and cryo-EM maps reported in this work have been deposited in the PDB (https://www.rcsb.org/) and Electron Microscopy Data Bank (EMDB, https://www.ebi.ac.uk/emdb/).

Cryo-EM structure of a human flippase mutant ATP11C Q79E-CDC50A in PtdCho-occluded E2-AlF state: 9VKG; EMD-65136.

Cryo-EM structure of a human flippase mutant ATP11C Q79E-CDC50A in PtdSer-occluded E2-AlF state: 9VNT; EMD-65217.

Cryo-EM structure of a human flippase mutant ATP11C Q79E-CDC50A in PtdCho-open E2-BeF state: 9VSL; EMD-65302.

Cryo-EM structure of a human flippase mutant ATP11C Q79A-CDC50A in PtdSer-occluded E2-AlF state: 9VQ2; EMD-65258.

## Supporting information

This article contains supporting information.

## Conflict of interest

The authors declare that they have no conflicts of interest with the contents of this article.

## Supporting information

Supportinginformation
