# Supplementary material for: Cryo-EM structure of the ATP11C Q79E mutant reveals the structural basis for altered Phospholipid recognition
Source: J Biol Chem. 2025 Nov 12;302(1):110935. doi: 10.1016/j.jbc.2025.110935 (PMC12723151; doi:10.1016/j.jbc.2025.110935)
Supplement: Supportinginformation [file mmc1.pdf]

Supporting information for

Cryo-EM Structure of the ATP11C Q79E Mutant Reveals the Structural Basis for Altered Phospholipid Recognition

Yuheng Qian[1,2], Chai C. Gopalasingam[1], Christoph Gerle[3], Hideki Shigematsu[4], Kazuhiro Abe[1*], Atsunori Oshima[2,5,6,7,8*]

[1]Department of Chemistry, Faculty of Science, Hokkaido University, Japan

[2]Graduate School of Pharmaceutical Sciences, Nagoya University, Nagoya, Japan

[3]RIKEN SPring-8 Center, Kouto, Sayo-gun, Hyogo 679-5148, Japan

[4]Japan Synchrotron Radiation Research Institute (JASRI), SPring-8, 1-1-1 Kouto, Sayo, Hyogo 679-5148, Japan

[5]Cellular and Structural Physiology Institute (CeSPI), Nagoya University, Furo-cho, Chikusa-ku, Nagoya 461-8601, Japan

[6]Institute for Glyco-core Research (iGCORE), Nagoya University, Nagoya,

Aichi 464-0814, Japan

[7]Center for One Medicine Innovative Translational Research (COMIT), Gifu

University Institute for Advanced Study, Gifu, 501-11193, Japan

[8]Research Institute for Quantum and Chemical Innovation, Institutes of

Innovation for Future Society, Nagoya University, Furo-cho, Chikusa-ku,

Nagoya 461-8601, Japan

Keywords: P-type ATPase, flippase, P4-ATPase, membrane protein, cryo-electron microscopy, phospholipid, transporter

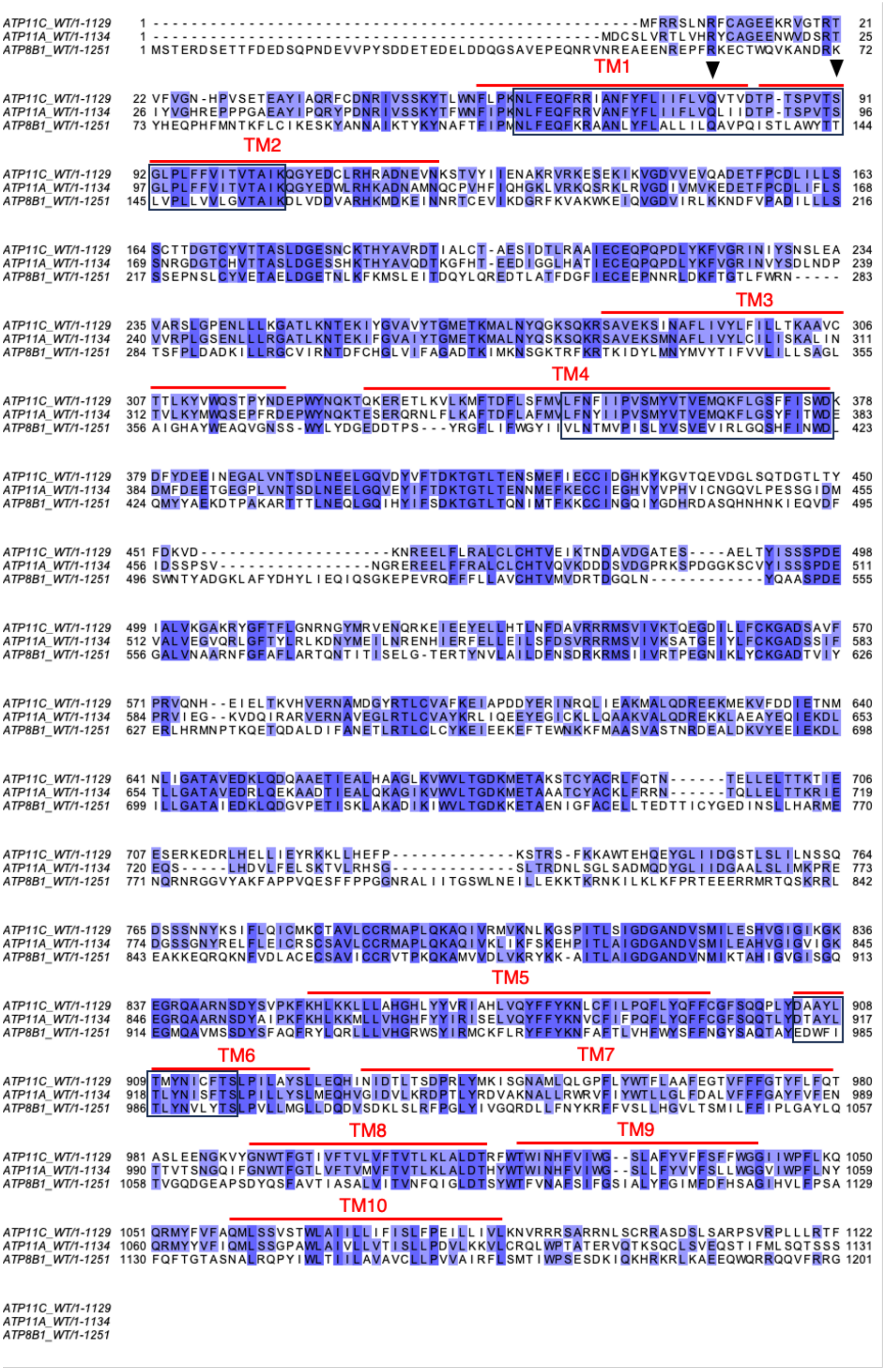

Fig. S1I Sequence alignment of ATP11C and ATP11A and ATP8B1.

The full-length amino acid sequences were aligned using CLUSTALW and visualized in Jalview (Uniprot ID: hATP8B1(O43520); hATP11A (P98196); hATP11C (A0A804HIW2). Residues are color-coded based on percentage identity: darker blue indicates higher conservation. Arrows show the Q79 position and S91 position in ATP11C. Black framed areas indicate the lipid binding cavity regions.

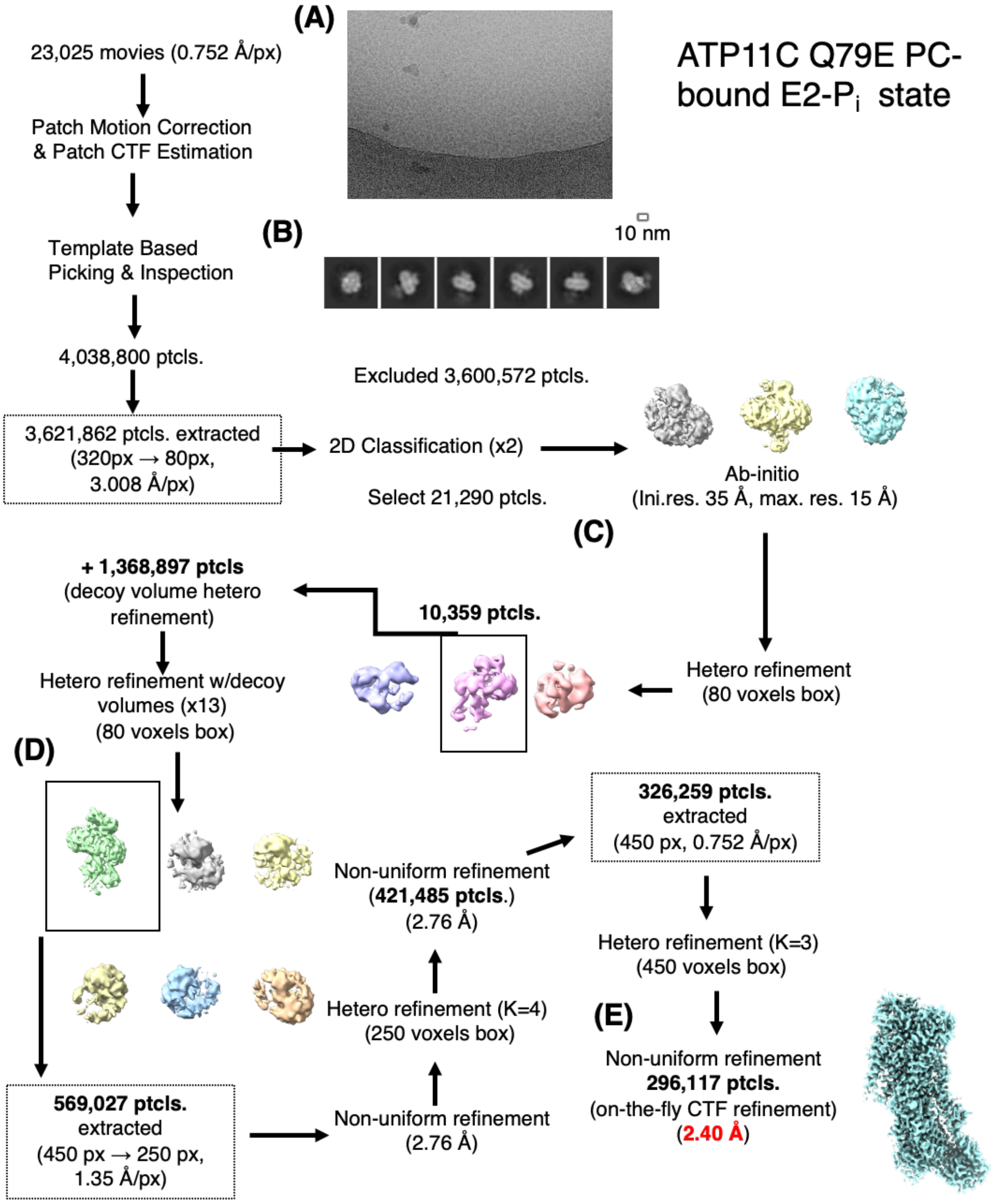

Fig. S2I Cryo-EM data processing workflow in cryoSPARC.

All datasets in this study were processed using a similar workflow in cryoSPARC. Shown here is the representative workflow for ATP11C Q79E in the PC-bound E2-P_i state. A, Representative micrograph. Scale bar in lower right. B, 2D templates employed for template based picking. C, Ab initio reconstructions of selected particles from 2D classification. After one round of heterorefinement, a further ~1.3 million particles were added for extensive rounds of heterorefinement. D, ~569,000 particles were re-extracted at 1.35 Å/px before non-uniform refinement, heterorefinement and non-uniform refinement, before re-extraction at 450 px. E, After hetero refinement and a non-uniform refinement, a 2.40 Å reconstruction was obtained. Right, density map (cyan) of the final reconstruction.

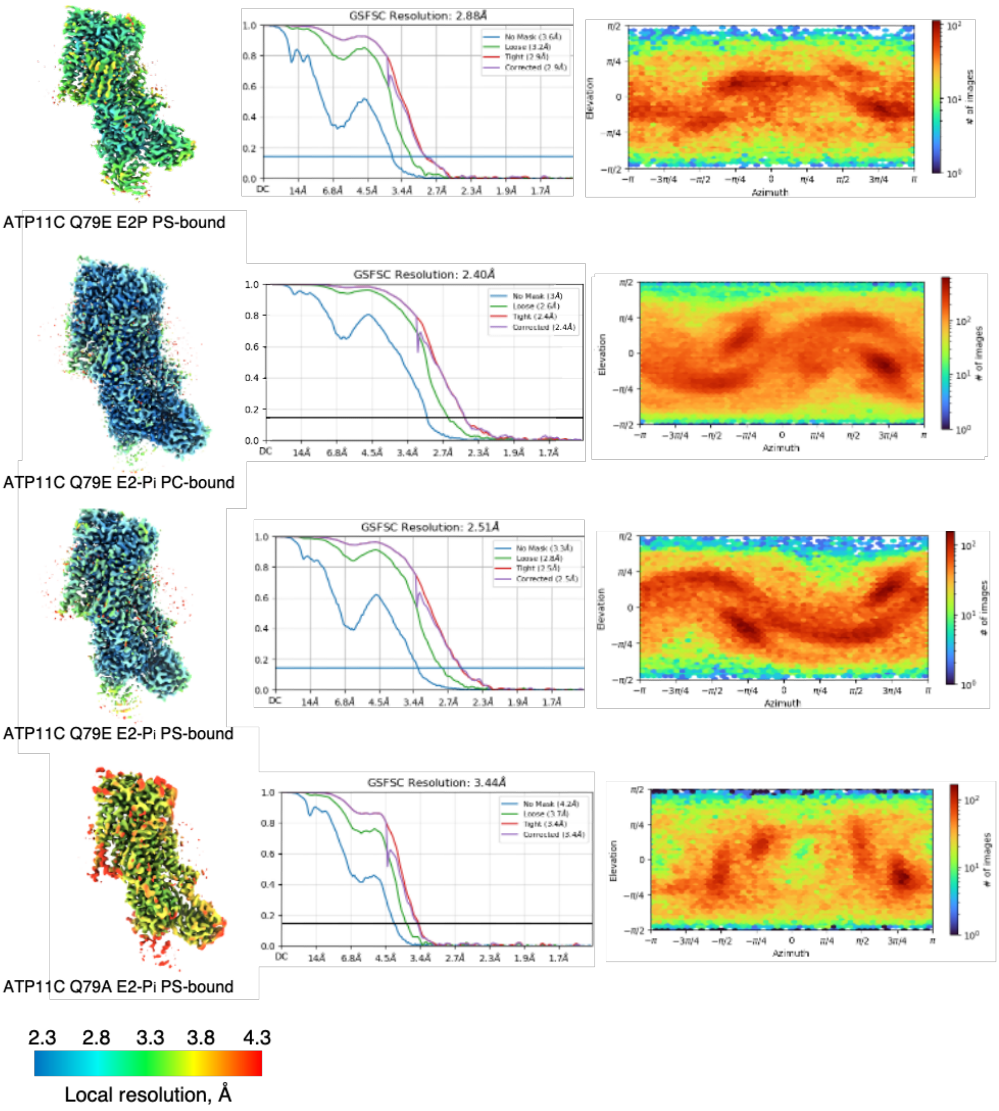

Fig. S3l Summary of cryo-EM analysis

Unsharpened maps calculated by cryoSPARC (left), Fourier Shell Correlation (FSC) plots used for resolution estimation (center) and angular distribution plots of the particles included in the 3D reconstruction (right) are shown for the indicated dataset. Color scale for the local resolution is shown in the lower left of the figure.

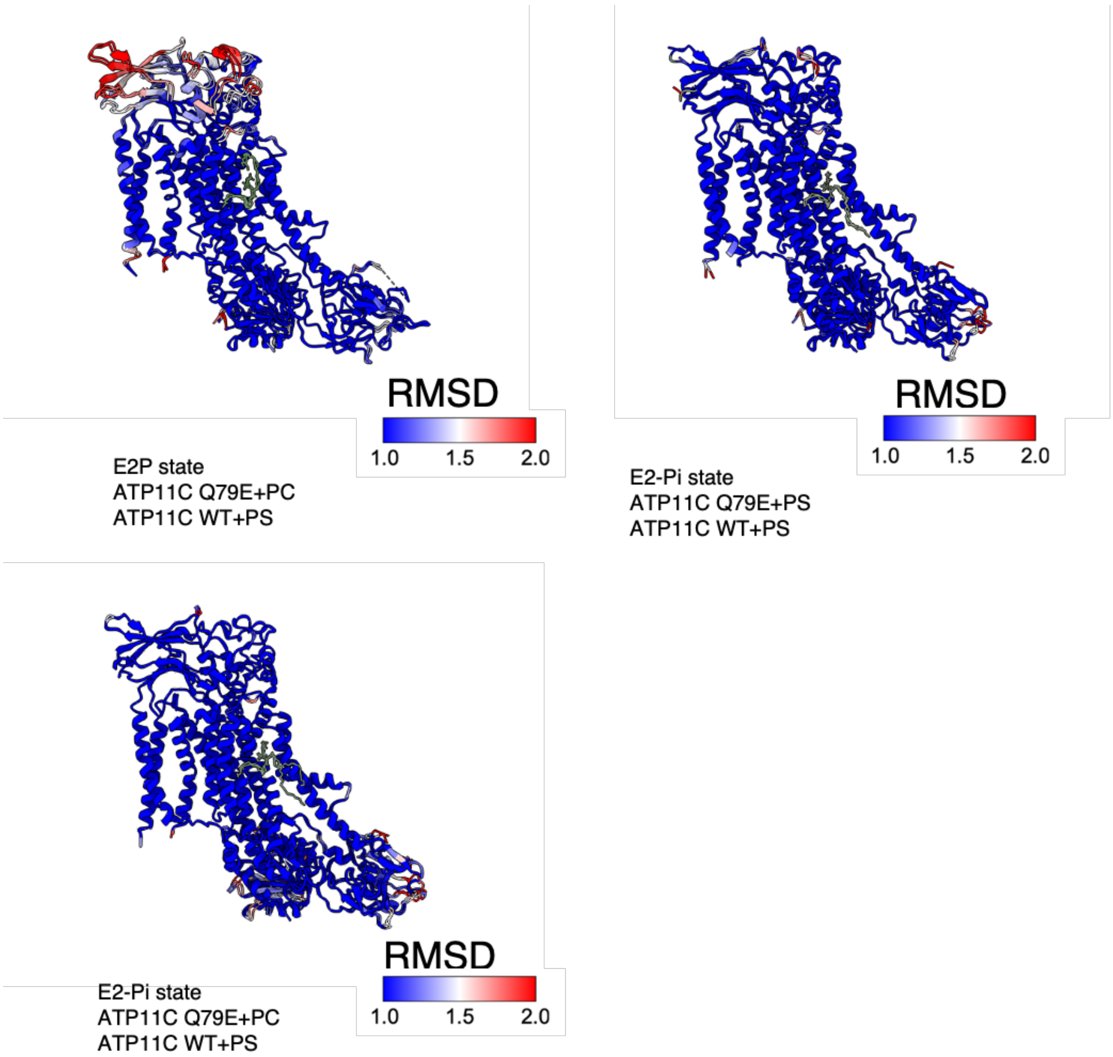

Fig. S4I Structural comparison of ATP11C WT and Q79E mutant colored by RMSD. Structures were superimposed, and the root-mean-square deviation (RMSD) values of corresponding Cα atoms were calculated. The structures are colored according to RMSD: blue indicates regions with low RMSD (high structural similarity).

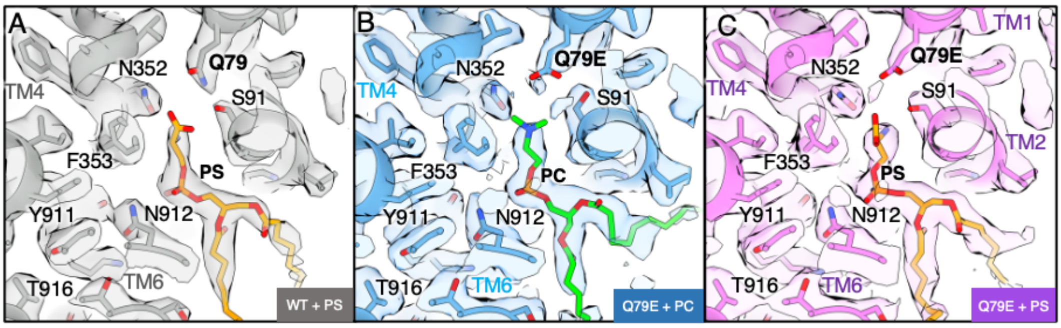

Fig. S5I Close-up view of phospholipid binding site of E2-P_i state, cryo-EM density maps are fitted with PDBs for WT (A, PDB: 7BSV), Q79E mutant with PC (B) and Q79E with PS (C).

Table S1l Statistics of the structural analysis

	Cryo-EM structure of a human flippase mutant ATP11C Q79E-CDC50A in PtdCho-occluded E2-Pi state	Cryo-EM structure of a human flippase mutant ATP11C Q79E-CDC50A in PtdSer-occluded E2Pi state	Cryo-EM structure of a human flippase mutant ATP11C Q79A-CDC50A in PtdSer-occluded E2-Pi state	Cryo-EM structure of a human flippase mutant ATP11C Q79E-CDC50A in PtdCho-bound E2P state
PDB ID	9VKG	9VNT	9VQ2	9VSL
EMDB	EMD-65136	EMD-65217	EMD-65258	EMD-65302
**Data collection**				
Magnification	60,000			
Voltage (kV)	300			
Electron exposure (e⁻/Å²)	60			
Defocus range (μm)	0.8-1.8			
Pixel size （Å/pix）	0.752			
Symmery imposed	C1			
Movies (no.)	23,350	5,550	7,148	8,950
Initial particles (no.)	4,038,800	3,082,297	3,956,404	2,868,613
Final particles (no.)	296,117	105,729	84,218	69,205
Box size (extract/final, pix)	320/450	336/450	320/450	336/450
Map resolution (Å)	2.40	2.51	3.44	2.88
FSC threshold	0.143			
**Refinement**				
initial model used (PDB)	7BSV	7BSV	7BSV	7BSU
Model resolution (Å)	3.00			3.20
FSC threshold	0.5			
Model composition				
Non-hydrogen	9,282	9,153	9,280	9,248
Protein residues	1139	1125	1139	1137
Ligands	ALF, MG, NAG, MAN, PCW	ALF, MG, NAG, MAN, P5S	ALF, MG, NAG, MAN, P5S	BEF, MG, NAG, PCW
B-factor (mean value, Å²)				
Protein	37.27	51.9	52.38	50.53
Ligand	58.13	53.88	61.87	45.77
Water	6.61	30.43	30.94	0
R.m.s. deviations				
Bond length (Å)	0.003	0.004	0.003	0.003
Bond angles (°)	0.623	0.781	0.724	0.704
Validation				
MolProbity score	1.84	2.02	1.76	2.15
Clashscore	6.58	8.15	8.31	10.00
**Ramachandran Plot**				
Favored (%)	96	96	95	95
Allowed (%)	4	4	5	5
Disallowed (%)	0.00	0.00	0.00	0.00
